# Ephrin-B3 coordinates timed axon targeting and amygdala spinogenesis for innate fear behaviour

**DOI:** 10.1038/ncomms11096

**Published:** 2016-03-24

**Authors:** Xiao-Na Zhu, Xian-Dong Liu, Suya Sun, Hanyi Zhuang, Jing-Yu Yang, Mark Henkemeyer, Nan-Jie Xu

**Affiliations:** 1 Department of Neurology, Institute of Neurology, Ruijin Hospital, Shanghai Jiao Tong University School of Medicine, 200025 Shanghai, China; 2 Department of Anatomy, Histology and Embryology, Neuroscience Division, Shanghai Jiao Tong University School of Medicine, 200025 Shanghai, China; 3 Department of Pharmacology, Shenyang Pharmaceutical University, 110016 Shenyang, China; 4 Department of Biochemistry and Molecular Cell Biology, Shanghai Key Laboratory for Tumor Microenvironment and Inflammation, Shanghai Jiao Tong University School of Medicine, 200025 Shanghai, China; 5 The Institute of Health Sciences, Shanghai Institues for Biological Sciences, Chinese Academy of Sciences/Shanghai Jiao Tong University School of medicine, 200025 Shanghai, China; 6 Departments of Neuroscience and Developmental Biology, Kent Waldrep Center for Basic Research on Nerve Growth and Regeneration, University of Texas Southwestern Medical Center, Dallas, 75390 Texas, USA

## Abstract

Innate emotion response to environmental stimuli is a fundamental brain function that is controlled by specific neural circuits. Dysfunction of early emotional circuits may lead to neurodevelopmental disorders such as autism and schizophrenia. However, how the functional circuits are formed to prime initial emotional behaviours remain elusive. We reveal here using gene-targeted mutations an essential role for ephrin-B3 ligand-like activity in the development of innate fear in the neonatal brain. We further demonstrate that ephrin-B3 controls axon targeting and coordinates spinogenesis and neuronal activity within the amygdala. The morphological and behavioural abnormalities in ephrin-B3 mutant mice are rescued by conditional knock-in of wild-type ephrin-B3 during the critical period when axon targeting and fear responses are initiated. Our results thus define a key axonal molecule that participates in the wiring of amygdala circuits and helps bring about fear emotion during the important adolescence period.

## Abstract (web summary)

The molecular mechanism underlying initial circuit wiring in amygdala is poorly understood. Here the authors show that ephrin-B3 is required for axon targeting and amygdala spinogenesis during a critical period in development, and plays an important role in amygdala mediated fear responses.

## Graphical abstract

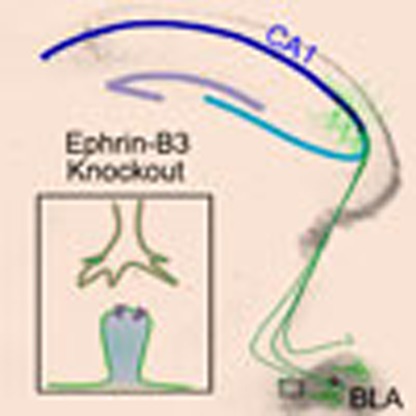

Mammalian evolution has required the successful development of emotional systems to cope with various environmental stimuli^1^,^2^. Amygdala serves as the core of the brain emotional system that primes innate defensive reactions and learned fear behaviours^3^,^4^,^5^. In view of its widespread functional connections with sensory associative areas, the amygdala is regarded as a sensory gateway for integration of a wide array of emotional information^4^,^6^,^7^,^8^,^9^,^10^,^11^,^12^,^13^. In addition to emotional control, the amygdala participates in psychiatric disorders and, in particular, the socio-emotional impairment^14^. In human patients and animal models of neurodevelopmental disorders, including autism spectrum disorders and schizophrenia, dysfunction of amygdala-associated brain networks has been reported^14^,^15^,^16^,^17^,^18^. Determining the precise timing and mechanisms that control nerve wiring to form the amygdala network is crucial to our understanding of the plasticity of neuronal responses, and thus the molecular basis of emotional behaviour.

As the brain develops, there exist critical periods in which specific circuits are susceptible to environmental stimuli that participate in shaping certain types of innate behaviours or neural functions^19^,^20^. One critical period of nerve wiring involves various brain regions/nuclei that connect to the amygdala, leading to synapse formation and the generation of emotional arousal. We hypothesize that the inter-nucleus wiring is mediated by trans-synaptic interaction of guidance molecules with the following characteristics: (i) timed expression in associated essential nuclei, (ii) involved in synaptic formation/remodelling and (iii) crucial for neurotransmission and plasticity. Among the molecules fulfilling these criteria, ephrin and Eph receptors are involved in sensory integration and cognitive function and transduce bidirectional signals to integrate pre- and post-synaptic development on axon-dendrite/cell contact^21^,^22^,^23^. Our previous studies revealed essential roles for the transmembrane protein ephrin-B3 (henceforth referred to as eB3), a major member enriched in neurons of the cortex and hippocampus, and in axon pruning, synaptogenesis and synaptic plasticity, during early postnatal development^24^,^25^,^26^,^27^. The time-restricted roles of eB3 suggest its significance in neural circuit formation and initial inter-nucleus coordination necessary for the formation of key neural networks. This is in agreement with recent reports that link ephrin-B-EphB signal deficit to anxiety disorders^28^, autism^29^ and mental retardation^30^,^31^. However, whether eB3 plays a role *in vivo* in regulating emotional brain function remains unknown.

In the present study, we identify an early onset time for initial defensive behaviour, a specific response mediated by the activation of neurons in the amygdala, and demonstrate that synaptic eB3 is required for initial formation of primitive brain emotions. Furthermore, we find that eB3 serves as a major mediator for targeting of hippocampal CA1 axons into the amygdala and plays a trans-nucleus role in timed coordination of spinogenesis. The coordination of axon/synapse development and neuronal function is mediated by axonal eB3 that initiates trans-synaptic signals into amygdala neurons during the critical period in the adolescence brain when innate fears are initially formed. Our findings thus provide a molecular mechanism for how neural circuit assembly is processed and regulated to affect neuronal activity and innate fear behaviour.

## Results

### eB3 is required for amygdala-mediated fear responses

**Figure 1:**
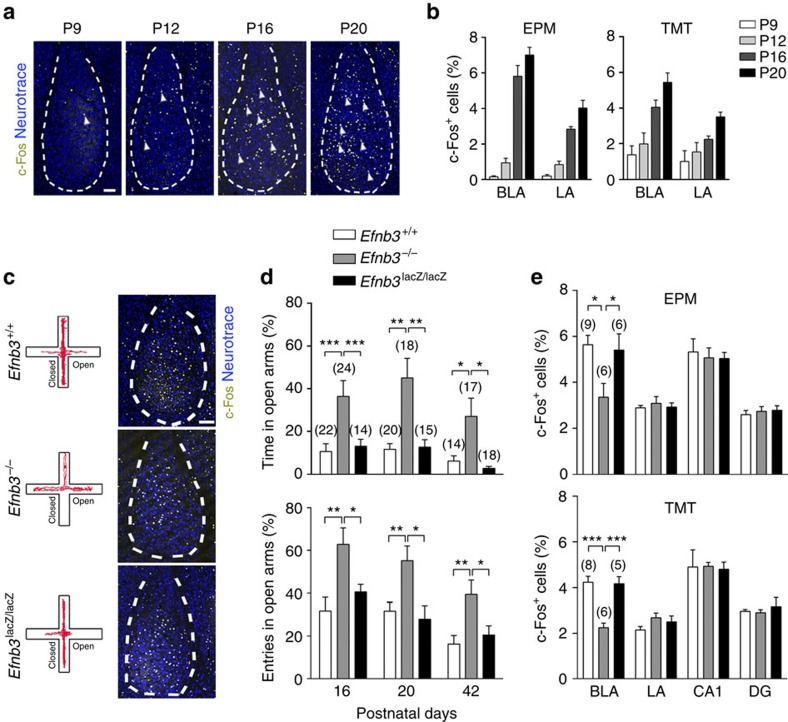
eB3 is required for defensive responses and activation of neurons in the amygdala. (**a**) c-Fos expression (arrowheads indicated yellow cells) in neurons of the amygdala was markedly elicited after an EPM behavioural trial from P9 to P20. Blue: neurotrace to stain BLA cell layer at P9–P20. Scale bar, 100 μm. (**b**) Quantification of c-Fos-positive cells (percentage of total neurotrace-positive cells) in BLA or lateral amygdala (LA) from P9 to P20 following an EPM or TMT behavioural trial. *n*=6 mice for each group. (**c**) Representative animal track of EPM, and representative images showing the c-Fos-positive cells in amygdala of *Efnb3*^+/+^, *Efnb3*^*−/−*^ and *Efnb3*^*lacZ/lacZ*^ mutant mice following EPM trial at P16. Scale bars, 100 μm. (**d**) *Efnb3*^−/−^, but not *Efnb3*^lacZ/lacZ^, mutant mice showed a longer time and a higher probability of open-arm entry than *Efnb3*^+/+^ at P16, P20 and P42. The experiments were triplicated independently. *n* values of mice used are indicated above group bars. *P<0.05; **P<0.01; ***P<0.001, one-way ANOVA. (**e**) Quantification of the cells with c-Fos expression at *Efnb3*^+/+^, *Efnb3*^−/−^ and *Efnb3*^lacZ/lacZ^ mutant in BLA, LA, CA1 and DG following an EPM or TMT trial. *n* values of mice are indicated above group bars. *P<0.05; ***P<0.001, one-way ANOVA.

To identify the initial onset of innate emotional behaviour, we combined a behavioural test with analysis of activated c-Fos expression in the amygdala to study defensive behavioural and neuronal reactions that respond to aversive stimuli. Using either a testing trial with an elevated plus maze (EPM)^32^,^33^,^34^ or an exposing trial to a predator odour TMT (2,5-dihydro-2,4,5-trimethylthiazoline)^35^,^36^,^37^, innate defensive responses can be triggered in juvenile or adult animals. In both behavioural paradigms, defensive responses were elicited specifically by threat stimuli in juvenile mice (Supplementary Figs 1 and 2). In these behavioural trials, a marked increase in number of c-Fos-positive cells was observed in several brain regions involved in sensory integration, the cortex and hippocampus, and in emotional signal processing, habenula nucleus, mediodorsal thalamic nucleus and ventromedial hypothalamus, during postnatal development (Supplementary Fig. 1c). In particular, c-Fos activation in the basolateral amygdala (BLA) was markedly increased starting from postnatal day 16 (P16; Fig. 1a,b).

Using gene-targeted mice we examined the potential roles for eB3 in BLA neuronal activity and innate behaviours with both behavioural paradigms. The *Efnb3*^−/−^ null mutant mice were subjected to EPM, and significant longer exploring time and more entries in the open arm of the maze were clearly observed from P16 until P42, reflecting a defect of innate fear response (Fig. 1c,d, Supplementary Movies 1 and 2). In TMT paradigm, *Efnb3*^−/−^ mice showed less avoidance responses compared with wild-type mice (Supplementary Fig. 2, Supplementary Movies 3 and 4). The altered fear responses were also reflected by a reduction in c-Fos-positive BLA neurons in either EPM or TMT paradigm (Fig. 1c,e). Furthermore, c-Fos-positive neurons were reduced in the habenula nucleus, mediodorsal thalamic nucleus and ventromedial hypothalamus of *Efnb3*^−/−^mice (Supplementary Fig. 1c). As a transmembrane protein, eB3 is capable of transducing bidirectional signals by either serving as ligand to bind and stimulate EphB receptor-expressing cells (forward signalling), or functioning as receptors *per se* to transduce autonomous reverse signals into the cells it is expressed on (reverse signalling), as demonstrated to be essential for dendritic development of hippocampal CA1 pyramidal neurons^27^. Therefore, to examine whether reverse signalling mediated by the cytoplasmic domain of eB3 is involved in innate behaviour, *Efnb3*^lacZ/lacZ^ mice were subjected to EPM and TMT paradigms. These eB3 mutant mice express a truncated ephrin-B-β-gal fusion protein (eB3-β-gal) that retains the extracellular and transmembrane domains to provide ligand-like activity but are unable to transduce reverse signals^38^. Unlike the *Efnb3*^−/−^ knockout, *Efnb3*^lacZ/lacZ^ mice showed no difference compared with the wild-type littermates in EPM or TMT fear responses (Fig. 1c,d), nor did they show reduction in c-Fos-positive BLA neurons (Fig. 1e). These genetic data indicate that eB3 likely serves a ligand-like role to stimulate forward signalling events needed for trans-nucleus wiring of the amygdala and formation of innate fear responses.

### eB3 mediates axon targeting and amygdala spinogenesis

**Figure 2:**
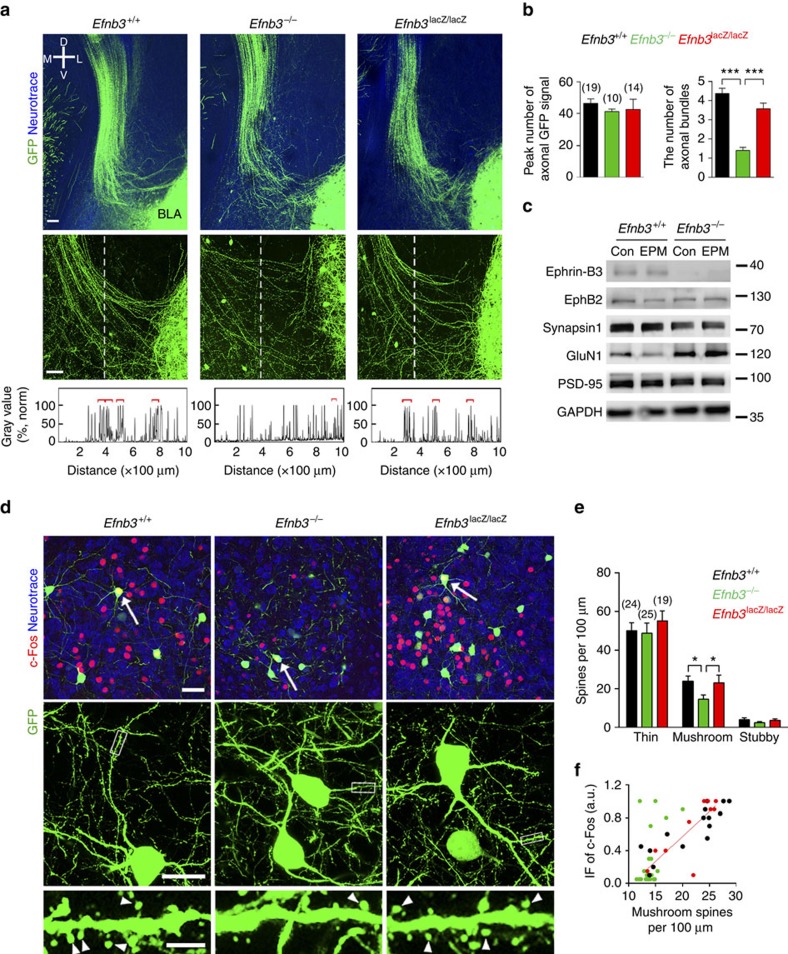
eB3 is required for axon targeting and spinogenesis in amygdala neurons. (**a**) Comparison of axonal bundles projected into amygdala between *Efnb3*^+/+^, *Efnb3*^−/−^ and *Efnb3*^*lacZ/lacZ*^ mice with Thy1-GFP M background. The vertical dotted line crossing the axon bundles is 1,000-μm long from top to bottom and 300 μm distance from left lateral edge of BLA. Scale bars, 100 μm. (**b**) Quantification of the total peak number of axonal signals with GFP crossing the dotted line and fasciculated axonal bundles (indicated with red square brackets). *n* values of brain slices from 6 to 7 mice per group are indicated above each group bar. ***P<0.001, one-way ANOVA. (**c**) Protein expression of synaptic molecules in amygdala of *Efnb3*^+/+^ and *Efnb3*^−/−^ mice. Synapsin1 expression is downregulated, while GluN1 (NMDA receptor) is upregulated in *Efnb3*^−/−^ mice. (**d**) *Efnb3*^−/−^ but not *Efnb3*^lacZ/lacZ^ mutant mice showed delayed spine maturation and reduced c-Fos activation in BLA neurons at P16. Red is c-Fos immunofluorescence and green is the small number of random BLA neurons that are Thy1-GFP M positive and outline post-synaptic spine structures. Scale bars, 40 μm, 25 μm and 5 μm for the upper, middle and lower panels, respectively. (**e**) Quantification of density for various types of spines in WT, *Efnb3*^*−/−*^ and *Efnb3*^lacZ/lacZ^ mutant at P16. *n* values of neurons from 5 to 6 mice per group are indicated above each group bar. *P<0.05, one-way ANOVA. (**f**) Correlation of the c-Fos immunofluorescence (IF) and the density of mushroom spines in c-Fos-positive neurons (left panel) in *Efnb3*^+/+^, *Efnb3*^*−/−*^ and *Efnb3*^lacZ/lacZ^ mutant at P16 following EPM trial. *n*=15 neurons from 6 mice for *Efnb3*^+/+^, *r*^2^=0.75; *n*=22 neurons from 8 mice for *Efnb3*^*−/−*^, *r*^2^=0.24; *n*=10 neurons from 5 mice for *Efnb3*^lacZ/lacZ^, *r*^2^=0.63. All the samples were from triplicated experiments.

By using *in situ* hybridization, eB3 transcripts are highly detected in the forebrain, and in particular the level of eB3 in hippocampal CA1 region reaches its peak at P14 (Supplementary Fig. 3a), a time that parallels the onset of initial defensive behaviour. We were then curious about how loss of eB3 caused such behavioural defects in EPM and TMT paradigms. We first examined for morphological abnormalities at P16 in *Efnb3*^−/−^ mutant mice that also contain the Thy1-GFP M transgenic line, which visualizes a subset of neurons during the postnatal critical period^39^. We used two-photon images from 400-μm-thick coronal brain sections to identify fluorescent long projecting axons and observed a bundle of downward-projecting fibres starting from the ipsilateral edge of the hippocampus and extending into the amygdala (Fig. 2a). The upper part of the axon bundles look similar between mutant and wild type, exhibiting a thick fasciculated track, indicating that eB3 deletion did not affect the overall axon growth/guidance and projection from the hippocampus. However, in *Efnb3*^−/−^ mutant we found that in the more distal region of the bundle, as the axon terminals enter the amygdala, the nerve fibres were loosely packed and not well-wired with BLA neurons in contrast to the tightly and neatly fasciculated bundles observed in the wild-type and *Efnb3*^lacZ/lacZ^ mice (Fig. 2a,b). The abnormalities were not likely attributable to the changes in axon growth as mentioned above or neurogenesis (Supplementary Fig. 4). This result indicates that the extracellular segment rather than the intracellular domain of eB3 is required for normal fasciculation and targeting of these hippocampal neuron axon projections into the BLA. We thus dissected the amygdala tissue out and examined it for changes in synaptic molecules in the BLA of wild-type and *Efnb3*^−/−^ mutant mice at P16. We observed an obvious decrease in the pre-synaptic marker synapsin and an adaptive increase of post-synaptic calcium channel GluN1 (Fig. 2c). Altogether, these results suggest that the loss of eB3 expression results in abnormal targeting of hippocampal axons extending into BLA, and that this affects normal pre- and post-synaptic integration.

Notably, eB3 is not expressed in BLA neurons as indicated by X-gal staining in *Efnb3*^lacZ^ mice (Supplementary Fig. 3), in which the eB3-β-gal fusion protein is expressed in the correct temporal, spatial and subcellular pattern as the endogenous wild-type protein and provides for a high signal-to-noise ratio reporter^27^. We thus hypothesized that eB3 expressed on hippocampal CA1 axons that target the amygdala may affect BLA neurons by binding with one of its receptors EphB2, which is expressed in BLA^28^, to control targeting of pre-synaptic axon terminals and initiate synapse formation. *EphB2*^−/−^ mice resembled *Efnb3*^−/−^ innate fear behaviours in EPM paradigm (Supplementary Fig. 5a), supporting a trans-nucleus eB3-EphB2 interaction. As the combined parameters of spine density and spine size/shape determine the total excitatory input from these pre-synaptic terminals^40^,^41^,^42^, we analysed spine morphogenesis of BLA neurons by using confocal images of brain sections from *Efnb3*^−/−^ mutant mice to examine the possible trans-nucleus effect of eB3. According to the algorithm for classification of spine shape adapted in our previous study^43^, we analysed the density of various spine groups in Thy1-GFP-labelled BLA neurons, and observed a significant reduction in the fraction of mature mushroom spines but no obvious change in the fraction of thin or stubby spines in *Efnb3*^−/−^ mice at P16 when compared with wild-type or *Efnb3*^lacZ/lacZ^ mice (Fig. 2d,e). The decrease in mushroom spines in *Efnb3*^−/−^ mice persisted at P20 but reached a normal level at P42 (Supplementary Fig. 6). Consistently, *EphB2*^−/−^ mice also showed decreased mushroom spines in BLA neuron dendrites at P16 but with normal hippocampus projected axon bundles towards BLA (Supplementary Fig. 5b,c). To investigate the functional consequence of abnormal spine morphology, we analysed for c-Fos immunofluorescence in GFP-labelled amygdala neurons and plotted the c-Fos level of these neurons versus the density of mushroom spines. We found that the density of these mushroom spines was actively correlated with the c-Fos level in wild-type (*r*^2^=0.75, *P*<0.0001) and *Efnb3*^lacZ/lacZ^ mice (*r*^2^=0.63, *P*<0.0001), while this correlation was completely abolished in *Efnb3*^−/−^ mice (*r*^2^=0.24, *P*=0.02; Fig. 2f). These results suggest that eB3 is required for the timely maturation of mushroom spines and robust neuronal activation (that is, c-Fos) in the amygdala at the early postnatal period.

### eB3 rescue initiates timed axon targeting and fear responses

**Figure 3:**
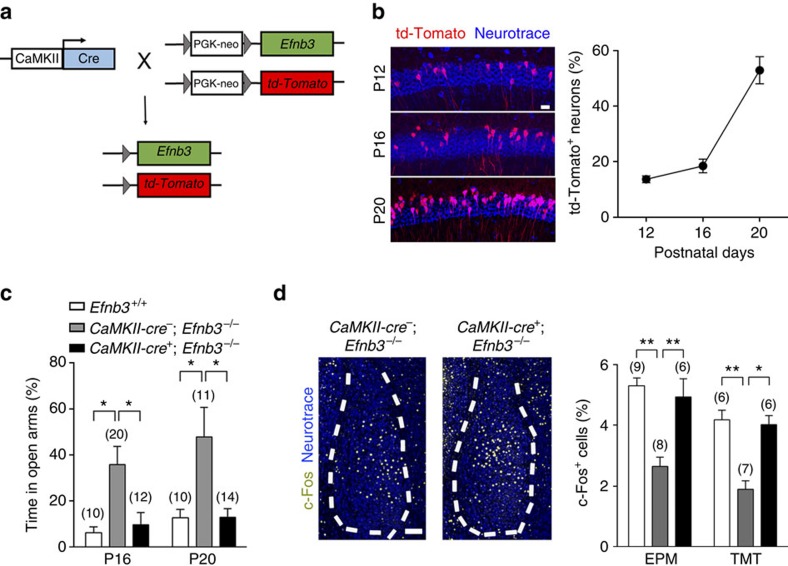
Spatial-specific rescue of eB3 restores defensive responses in *Efnb3*^−/−^ mutant. (**a**) Transgenic mice expressing *CaMKII-Cre* recombinase were crossed with *td-Tomato*^+^; *Efnb3*^−/−^ homozygotes. EB3 expression in the initial *td-Tomato*^+^; *Efnb3*^−/−^ mutant was restored and indicated with td-Tomato reporter on *Cre*-mediated excision of the *loxP*-flanked *PGK neo* cassette. (**b**) *CaMKII-cre* mediated recombination in CA1 area visualized with td-Tomato fluorescence was detected in P12, P16 and P20 hippocampal sections in *CaMKII-cre*^+^; *td-Tomato*^+^; *Efnb3*^−/−^ mice. Scale bar, 50 μm. The percentage of td-Tomato-positive cells in CA1 area increased gradually during postnatal development. *n*=3 mice for per group. (**c**) *CaMKII*-*cre*^+^; *Efnb3*^−/−^ mice showed a shorter time than *CaMKII*-*cre*^−^; *Efnb3*^−/−^ mice in open arms of EPM at P16. The experiments were triplicated independently. *n* values of mice used are indicated above group bars. *P<0.05, one-way ANOVA. (**d**) Representative images showing the c-Fos-positive cells in amygdala of *CaMKII*-*cre*^−^; *Efnb3*^−/−^ and *CaMKII*-*cre*^+^; *Efnb3*^−/−^ mice at P16. Scale bars, 100 μm. The percentage of c-Fos-positive cells in BLA of *CaMKII*-*cre*^+^; *Efnb3*^−/−^ mice was restored compared with that of *CaMKII*-*cre*^−^; *Efnb3*^−/−^ following an EPM or TMT behaviour trial at P16. *n* values of mice used are indicated above group bars. *P<0.05; **P<0.01; ***P<0.001, one-way ANOVA.

To further verify the temporal and spatial specificity of eB3 for defensive responses, we utilized a conditional knock-in strategy to induce eB3 re-expression at an endogenous level in the *Efnb3*^−/−^ mice. The *Efnb3*^−/−^ mutant line we used above was generated by inserting a *loxP*-flanked PGK-neo cassette into the intron between the fourth and the fifth exons of *Efnb3*, which results in a protein-null allele^27^. This insertion allows us to conditionally remove the floxed neo cassette by adding Cre recombinase and thereby rescue wild-type eB3 expression. To address the question about brain specificity for the role of eB3, we first crossed the *Efnb3*^−/−^ mutant with a CaMKII-Cre transgene^44^, a forebrain excitatory neuron-specific Cre for the PGK-neo cassette deletion, and a *loxP*-flanked Stop-tdTomato reporter (*CamKII-cre*^+^; *tdTomato*^+^; *Efnb3*^−/−^) to visualize the distribution and efficiency of CaMKII-Cre recombination (Fig. 3a). Our data showed that CaMKII-Cre recombination increased rapidly in the hippocampus CA1 area, where eB3 is highly expressed, during P12 to P20 (Fig. 3b). In the EPM trial at P16 we found that the defect in behaviour and c-Fos activation observed in *Efnb3*^−/−^ mutants was restored back to normal in *CamKII-cre*^+^; *tdTomato*^+^; *Efnb3*^−/−^ mice (Fig. 3c,d). These data rule out the possibility that the changes in the defensive responses observed in the *Efnb3*^−/−^ mice are the result of a possible defect due to loss of eB3 earlier in brain development before P12, and suggest that the role of eB3 is during the critical postnatal stages when fear behaviour initiates.

As adjoining nuclei, the hippocampus and amygdala are functionally associated with each other in the adult brain^8^,^45^,^46^,^47^,^48^, though clinical evidence of patients with focal lesions to these two systems indicate that they can also operate independently^7^,^8^,^49^. We validated the anatomical and functional connection between the two regions in juvenile mice by using both slice electrophysiology (Supplementary Fig. 7) and dye tracing (Supplementary Fig. 8). We recorded fEPSP signals in BLA following hippocampal electrode stimuli in the ventral hippocampal CA1 region, and observed constant fEPSP in slices from P16 wild-type mice that were significantly reduced in amplitude in the *Efnb3*^−/−^ mice (Supplementary Fig. 7). Through dye tracing from the hippocampal CA1 region, labelled axons were detected in BLA as early as P12 (Supplementary Fig. 8).

The role of eB3 was further examined using an injected AAV2-NLS-Cre-induced rescue strategy in the *Efnb3*^−/−^ mice. We injected AAV2-td-Tomato control or AAV2-NLS-Cre virus into the CA1 region of the intermediate or ventral hippocampus in newborn *tdTomato*^+^; *Efnb3*^−/−^ mice to trace axon projections (Supplementary Fig. 9a), and found that td-Tomato fluorescence-expressing axons appeared in the region of Thy1-GFP-labelled axon bundles, indicating the anatomical origin of these fibres (Supplementary Fig. 9b). The projected axon terminals from td-Tomato-labelled CA1 neurons were observed to form synapses with dendritic spines of BLA neurons (Supplementary Fig. 9c). At P16 these mice were subjected to behavioural tests, and a significant restoration in innate defensive response, amygdala c-Fos activation and mushroom spines was observed in AAV2-NLS-Cre-injected *td-Tomato*^+^; *Efnb3*^−/−^ mice, which was comparable to the normal level (Supplementary Fig. 9d–f). The ability of CA1-injected AAV2-NLS-Cre to rescue the *Efnb3*^−/−^ phenotypes is consistent with the idea that hippocampal eB3 is required for trans-nuclei wiring into the BLA.

**Figure 4:**
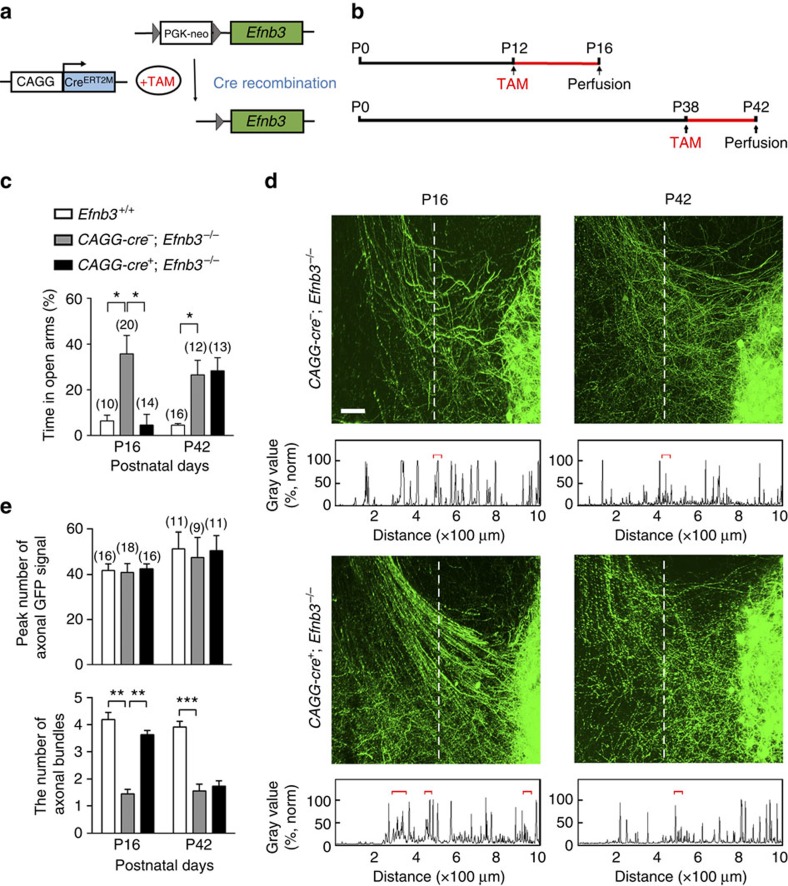
Temporal-specific rescue of eB3 restores defensive responses in *Efnb3*^−/−^ mutant. (**a**) Transgenic mice expressing tamoxifen-inducible Cre recombinase under the control of the CAGG promoter (*CAGG-Cre*^ERT2M^) were crossed with *Efnb3*^−/−^ homozygotes. EB3 expression in the initial *Efnb3*^−/−^ mutant is restored on tamoxifen administration to induce Cre-mediated excision of the *loxP*-flanked *PGK neo* cassette. (**b**) Schedule of tamoxifen treatment and brain perfusion during postnatal development. All the mice including wild-type controls were treated with tamoxifen injection at the indicated time. (**c**) *CAGG*-*Cre*^+^; *Efnb3*^−/−^ mice showed a shorter time in EPM open arms than *CAGG*-*Cre*^−^; *Efnb3*^−/−^ at P16 following tamoxifen treatment at P12 but not when tamoxifen was applied at P38. The experiments were triplicated independently. *n* values of mice used are indicated above group bars. *P<0.05, one-way ANOVA. (**d**) Representative images showing Thy1-GFP M-labelled axon bundles projecting into amygdala of *CAGG*-*cre*^−^; *Efnb3*^−/−^ and *CAGG*-*cre*^+^; *Efnb3*^−/−^ mice. Scale bars, 100 μm. (**e**) Quantification of the total peak number of axonal GFP signal crossing the dotted line and fasciculated axonal bundles (indicated with red square brackets). *n* values of brain slices from 5 to 6 mice per group are indicated above each group bar, **P<0.01; ***P<0.001, one-way ANOVA.

We next addressed whether the role of eB3 in trans-nucleus regulation was time dependent. We utilized *Efnb3*^−/−^ mice containing a ubiquitous *CAGG-Cre*^ERT2M^ transgene and the *loxP*-franked Stop td-Tomato reporter (*CAGG*-*cre*^+^; *Efnb3*^−/−^), and treated them with tamoxifen on the first day of postnatal period P12–P16 or P38–P42 to activate Cre recombinase and rescue eB3 expression (Fig. 4a,b and Supplementary Fig. 10). After tamoxifen treatment at P12, the defective defensive behaviour observed in *Efnb3*^−/−^ was restored to a normal level, comparable to that of wild-type littermates, in the end of the eB3 rescuing time P16 (Fig. 4c). However, tamoxifen treatment at P38 failed to rescue the behaviour, suggesting a time-restricted role for eB3 in generation of fear responses. We then asked whether the time-restricted modulation by eB3 involves time-dependent axon targeting and connection. Following tamoxifen treatment, we observed loosely packed and tangled axon bundles adjoining the amygdala at P16 or P42 in *CAGG*-*cre*^−^; *Efnb3*^−/−^ control mice that showed the behavioural defects in innate fear responses (Fig. 4d,e). However, the abnormal axonal morphology for Thy1-GFP-expressed axon bundles was restored in tamoxifen**-treated *CAGG*-*cre*^+^; *Efnb3*^−/−^ mutant mice of P12–16 rescuing period, which maintained normal appearance at P42 (Supplementary Fig. 11), but not in the mice of P38–P42 rescuing period (Fig. 4d,e). These data indicate time-restricted roles for eB3 in axon targeting and neuronal connection to control defensive behaviour.

### Timed trans-nucleus coordination for functional spinogenesis

**Figure 5:**
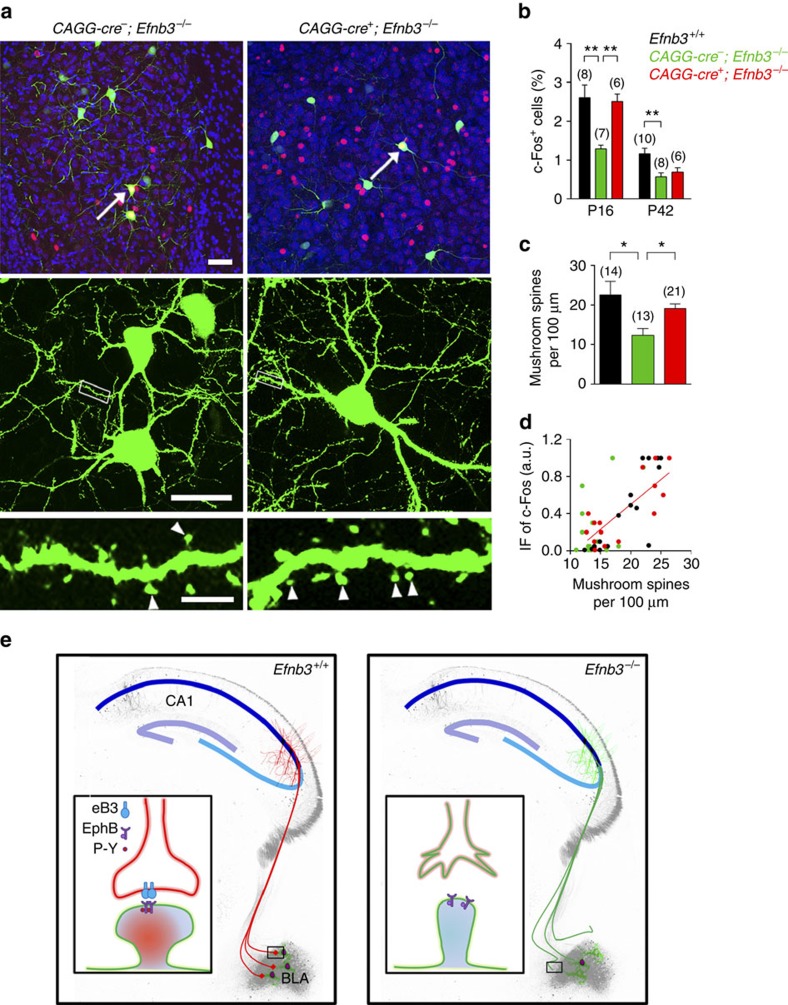
Time-specific eB3 rescues synaptogenesis and defensive responses in *Efnb3*^−/−^ mutant. (**a**) eB3 rescue improved spine maturation and c-Fos activation in BLA neurons at P16 following tamoxifen administration at P12 in *CAGG*-*Cre*^+^; *Efnb3*^−/−^ mice. Scale bars, 40 μm, 25 μm and 5 μm for the upper, middle and lower panels, respectively. (**b**) The percentage of c-Fos-positive cells in BLA in *CAGG*-*Cre*^+^; *Efnb3*^−/−^ mice after an EPM trial at P16 increased significantly compared with *CAGG*-*Cre*^−^; *Efnb3*^−/−^ mice following tamoxifen treatment at P12 but not that at P38. *n* values of mice used are indicated above group bars. **P<0.01, one-way ANOVA. (**c**) eB3 rescue increased the density of mushroom spines in BLA neurons at P16 following tamoxifen administration at P12 in *CAGG*-*Cre*^+^; *Efnb3*^−/−^ mice. *n* values of neurons from 5 to 6 mice each group are indicated above each group bar. *P<0.05, one-way ANOVA. (**d**) Correlation of c-Fos immunofluorescence (IF) and the density of mushroom spines in c-Fos-positive neurons (left panel) in *Efnb3*^+/+^, *CAGG*-*cre*^−^; *Efnb3*^−/−^ and *CAGG*-*cre*^+^; *Efnb3*^−/−^ mice after EPM trial at P16. *n*=18 neurons from 6 mice for *Efnb3*^+/+^, *r*^2^=0.74; *n*=19 neurons from 7 mice for *CAGG*-*Cre*^−^; *Efnb3*^−/−^, *r*^2^=0.23; *n*=17 neurons from 6 mice for *CAGG*-*Cre*^+^; *Efnb3*^−/−^, *r*^2^=0.65. All the samples were from triplicated experiments. (**e**) Model of timed pre- and post-synaptic coordination for inter-nucleus neuronal connection for defensive neuronal responses. Pre-synaptic eB3 guides hippocampal axon targeting to connect with amygdala neurons through binding with EphB receptors. The eB3-EphB tetramer formation and EphB receptor kinase activation consolidate the trans-nucleus neuronal connection that is required for spinogenesis and threat stimulated defensive responses in BLA neurons. The mushroom spinogenesis and defensive neuronal activity are disrupted in *Efnb3*^−/−^ mice.

Finally, to examine if the timely eB3 rescue is sufficient to restore the spinogenesis and defensive neuronal reaction in BLA, we measured BLA c-Fos cell number and further detected the c-Fos immunofluorescence level of Thy1-GFP cells in tamoxifen-treated *CAGG*-*cre*^+^; *Efnb3*^−/−^ and *CAGG*-*cre*^−^; *Efnb3*^−/−^ mice (Fig. 5a). We found that the defect in BLA c-Fos activation observed in eB3 null was restored in tamoxifen-treated *CAGG*-*cre*^+^; *Efnb3*^−/−^ mutant mice at P16 but not at P42 (Fig. 5b). We further detected the morphology of BLA neurons in these mice after tamoxifen treatment at P16, and plotted their immunofluorescence level versus the density of mushroom spines to quantify for correlation of spinogenesis and neuronal activation. These data showed that the correlation of c-Fos immunofluorescence to mushroom spine density in BLA neurons was restored in *CAGG*-*cre*^+^; *Efnb3*^−/−^ mice (*r*^2^=0.65, *P*<0.0001) to a level comparable to that of wild-type mice (Fig. 5c,d). This is in agreement with the restoration of defensive behaviour.

In summary, our experimental data reveal that deletion of eB3 leads to significant defects in innate fear behaviour (Fig. 1), which is associated with a disruption in neuronal connection with amygdala neurons (Fig. 2). The results are consistent with the idea that eB3 stimulates forward signalling to guide axon targeting for trans-nucleus modulation and thereby coordinates spinogenesis and neuronal activation in BLA (Figs 1 and 2). Moreover, we used a conditional knock-in strategy to rescue endogenous eB3 to investigate the spatial and temporal relationship of eB3-mediated axonal targeting and the behavioural defects (Figs 3 and 4). The defensive neuronal response and innate fear behaviour is directly related to the time-restricted CA1 axon targeting and BLA neuron spinogenesis, while eB3 rescue at a later period failed to restore BLA neuronal activation and defensive behaviour (Fig. 5). Taken together, our study indicates that eB3 participates in axon targeting and trans-nucleus integration associated with spinogenesis of BLA neurons in a time-dependent fashion that couples neuronal activation to aversive cues, initiating defensive neuronal responses for innate fear.

## Discussion

In the present study, we uncovered biological mechanisms regulating innate emotional responses. We found that eB3 deletion leads to a significant defect in innate fear behaviour, which is associated with a disruption in connections of amygdala neurons. Our data may help resolve the controversy in the field of emotions about whether there are innate emotion circuits in the brain, and strongly support the presumption that innate emotions are of a ‘natural kind' and are hard-wired into brain circuits by evolution^50^. We further revealed that eB3 functions to integrate pre- and post-synaptic structures and thereby coordinates axon targeting, spinogenesis and neuronal reactions in the amygdala. This study indicates a new function for eB3 *in vivo*, which is in contrast with the previous studies on roles of ephrin-B/EphB-mediated bidirectional signalling for axon guidance and synaptic plasticity within the brain^26^,^27^,^31^,^51^,^52^,^53^,^54^,^55^,^56^. Interestingly, our results show that only the ligand-like activity of eB3 is required for axon targeting, spinogenesis and innate fear (Figs 1 and 2). This rules out the repulsive effects of eB3 reverse signalling for stereotyped axon pruning, which is mediated by its intercellular domain interacting with cytoskeletal regulators^26^, and suggests an adhesive effect of eB3 mediated by its extracellular domain for consolidation of neuronal connection on axon–dendrite contact.

Our study also revealed a specific developmental period for trans-nucleus modulation on spinogenesis of amygdala neurons and their defensive reaction, which in the mouse occurs between P12 and P16. This period is critical for the spine-reaction coupling in the developing brain (Fig. 5). In addition to previous studies on the essential role of critical developmental periods in numerous brain functions^19^,^20^, our results add evidence that the timed spinogenesis in BLA neurons coupled to aversive response serves as a key neural mechanism accounting for critical period-based innate defensive behaviours.

The brain elicits innate defensive reactions by integrating sensory inputs with critical brain nuclei that are involved in the initiation and modulation of amygdala-mediated emotional responses^9^,^12^. The expression of eB3 is observed mainly in the hippocampus but not in the amygdala, raising the question of how eB3 in these areas initiates molecular signals to modulate amygada through wired trans-nucleus synapses. Mechanistically, on binding of eB3 extracellular domain to the pre-synaptic axon terminals, EphB receptor tyrosine kinases in the post-synaptic responsive spines could become clustered and activated (see the model in Fig. 5e). Phosphorylated EphB receptors can regulate GluN1 receptor-dependent influx of calcium, leading to synaptic function-related gene expression^28^,^53^,^57^,^58^, which eventually facilitates spine morphogenesis and hence neuronal reactions to aversive cues.

In contrast to previous studies on the adult hippocampus that imply functions for exchanging messages as a specific engram via existing contextual pathways to participate in sophisticated learned emotions^4^,^9^,^12^, the trans-nucleus role of eB3 during the critical period suggests a distinct modulation of the developing hippocampus that is capable of controlling amygdala maturation and facilitates generation of innate emotions through regulating axon targeting. Developmental defects of the hippocampus and amygdala lead to severe behavioural disturbances that have been found clinically in children with autism, fragile X syndrome and brain disorders accompanied with fear and anxiety disorders^59^,^60^,^61^,^62^. In view of the genetic and protein linkage of ephrin-B-EphB/EphA signal deficit to anxiety disorders^28^, autism^29^, William's syndrome^63^ and Angelman's syndrome^31^, our further analysis clarifies the mechanisms by which the structural and functional circuit assembly to the amygdala are coordinated in the developing brain and leads to an increased understanding of the molecular basis for emotion-associated brain functions and disorders.

## Methods

### Mice

*Efnb3*^−^ (ref. 27), *Efnb3*^lacZ^ (ref. 38), *EphB2*^−^ (ref. 52), *CaMKII-Cre*^+^ (ref. 44) and *CAGG-Cre*^+^ (ref. 64) knock-out and knock-in mice and genotyping methods have been described previously. The *Efnb3*^−/−^ mutation was generated by inserting a *loxP*-flanked PGK-neo cassette into the intron between the fourth and the fifth exons of *Efnb3* that results in a protein-null allele^27^. The primary targeted allele of *Efnb3*^−/−^ homozygote was crossed with *CamKII*-Cre line/*CAGG*-Cre mouse line and a tdTomato reporter mouse line. Endogenous level of eB3 in the initial *Efnb3*^−/−^ mutant was restored on Cre expression that induced excision of the loxP-flanked PGK-neo cassette. All the mice were crossed with the Thy1-GFP M transgenic mouse line. Consecutive backcrosses to the CD1 strain were performed to move the mutations to CD1 background. Half male and half female of the mice from P9 to P42 were used in the experiments as indicated in the ‘Results' section. All experiments involving mice were carried out in accordance with the US National Institutes of Health Guide for the Care and Use of Animals under an Institutional Animal Care and Use Committee approved protocol and Association for Assessment and Accreditation of Laboratory Animal Care approved Facility at the Shanghai Jiao Tong University School of Medicine.

### Elevated plus maze

All tests were conducted according to a previous study^65^. Mice were habituated to handling and transport from the colony room to the behavioural room for 3 days before behavioural tests were begun. The visual ability of mice was checked with a light-induced locomotory behaviour in a transparent testing chamber, and the time to first body turn away from a bright flashlight (2 Hz) at one end was recorded^66^. Mice were given 1 h to habituate after transport to the behavioural room before any tests were conducted. All apparatuses and testing chambers were cleaned with 75% ethyl alcohol wipes between animals.

The EPM apparatus was made of dark grey plastic and consisted of two open arms (30 × 7 × 0.25 cm) opposed to two enclosed arms (30 × 7 × 15 cm) elevated 60 cm from the floor. Animals were placed in the central area of the apparatus with their head facing an enclosed arm (test duration: 5 min). The test was performed in a sound-attenuated and temperature-controlled (23±1 °C) room illuminated by one 40-W fluorescent bulb placed 3 m above the apparatus. Digitized video recordings (30 frames per second) with EthoVision software (Noldus Information Technology, Leesburg, VA) were used for behavioural analysis. The average velocity and total arm entries were considered as an index of locomotor activity, whereas the percentage of time spent in open arms and the percentage of open-arm entries were used as innate fear indexes.

### Innate olfactory exposure trial

An odour-TMT test^67^ was adapted to evaluate innate fear, in which the front door of the exposure PVC tube (10 × 2 cm) was made out of parafilm with a cavity in the centre to enable mice to smell specific odours. Mice were initially habituated to the testing tube for 2 min, and were then exposed to water, isoamyl acetate, sesame oil or TMT (100 μM) that were placed in the cavity by cotton ball for 2 min in the test session. The escape frequency from the cotton ball that stained specific odour was an index to evaluate innate fear levels.

### Western immunoblots

For western immunoblot, amygdala samples from *Efnb3*^*+*/+^ and *Efnb3*^*−*/*−*^ mutants at P16 were dissected, as previously described^68^, with minor modification. The brains placed on the dorsal face were transversely sliced through the optic chiasm and through the mammillary bodies. An oblique cut running 1.0-mm deep from the ventral surface of the temporal lobe to the hypothalamic fissure liberated a block of tissue containing mainly the medial amygdala and neighbouring piriform cortex from each hemisphere. The tissue was homogenized and solubilized at 4 °C for 30 min in lysis buffer (1% CHAPS, 137 mM NaCl, 2.7 mM KCl, 4.3 mM Na_2_HPO_4_, 1.4 mM KH_2_PO_4_, pH 7.2, 5 mM EDTA, 5 mM EGTA, 1 mM PMSF, 50 mM NaF, 1 mM Na_3_VO_4_ and protease inhibitors). The total protein lysates were separated by SDS-PAGE and analysed by Western blotting with primary antibodies rabbit anti-eB3 (1:1,000, Invitrogen #34–3600), goat anti-EphB2 (1:500, R&D #P54763) and mouse anti-GluN1 (1:1,000, BD Pharmingen #556308). Images have been cropped for presentation in Fig. 2c. Full-size images are presented in Supplementary Fig. 12.

### Axon bundle morphology

Mice of different ages and genotypes (as indicated in the text) with Thyl-GFP transgenic background were intracardially perfused with ice-cold 4% paraformaldehyde solution in phosphate buffer (pH 7.4). Thick (400 μm) hippocampal sections from the fixed brains were obtained using a vibratome (Leica 1000S). The sections were analysed by two-photon imaging (Olympus PVMPE-RS) with 25 × lens. The *Z* interval was 3 μm. The axon bundles projected to amygdala were selected for taking images. The brain slices selected for image collection included intermediate or ventral hippocampus^69^ and amygadala nucleus.

For quantitative analysis for the pattern of axon bundles, ∼10–15 brain slices from 4 to 5 mice were analysed for each genotype and age sample. We measured and quantified those axonal signals with ImageJ software along a crossing dotted line at a 300 μm distance from mediate edge of BLA. The fluorescence intensity of GFP crossing the dotted line was used as index for the axons signal that was presented as peak of grey value. The number of peaks with ⩾20% of maximum peak value was quantified per brain slice. Axonal signals with at least four consecutive peaks with an interval of ≤15 μm were determined as one axonal bundle. Acquisition of the images as well as morphometric quantification was performed under ‘blinded' conditions.

### Immunofluorescence and X-gal staining

For immunofluorescence, mice were processed 3 h after EPM or TMT trial. Coronal brain slices containing the whole amygdala were sectioned to a 50-μm thickness, washed with PBS and then incubated for blocking with permable buffter (0.3% Triton-100 in PBS) containing 10% donkey serum for 45–60 min. The sections were incubated in the primary antibodies against c-Fos (1:500; Cell Signaling Technology #2250S) overnight at 4 °C, and then incubated with the secondary Alexa Fluor 555 (1:200; Life Technology) and Neurotrace (1:500; Molecular Probes #N-21483). Confocal images of Thy1-GFP-expressed neurons from P9 to P20 mice were obtained with sequential acquisition settings at the maximal resolution of the microscope (1,024 × 1,024 pixels). For analysis of c-Fos-positive cells, the percentage of c-Fos-positive cells in total neurotrace-positive cells was quantified with imageJ software. Five brain sections were collected for quantifying c-Fos- and neurotrace-positive cells per mouse.

For EdU staining, the Click-iT kit with Alexa-647 (Life Technology) was used according to the manufacturer's instructions. The mice per time point received an intraperitoneal injection of EdU at 100 mg kg^−g^. Mice were perfused 24 h later, then sectioned to 30-μm thickness and co-stained with doublecortin antibody (1:200; Santa Cruz #sc-8066) and NeuN antibody (1:400; Cell Signaling Technology #24307).

To quantify the shape of spine, a procedure was adapted from our previous study^43^. Approximately 15–30 neurons from 3 to 4 mice were analysed for each genotype and age sample. To classify the shape of neuronal spines in slices we used NeuronStudio software package and an algorithm from ref. 70 with the following cutoff values: AR_thin_(crit)_=2.5, HNR_(crit)_=1.3, HD_(crit)_=0.4 μm. The protrusions with length 0.2–3.0 μm and Max width 3 μm were counted. For quantitative analysis for the strength of linear dependence between c-Fos immunofluorescence and the percentage of the spines with indicated heard diameter in BLA, each double-labelled neuron (Thy1-GFP and c-Fos) located in BLA was counted as a single data point. Acquisition of the images as well as morphometric quantification was performed under ‘blinded' conditions.

To detect the β-gal expression by X-gal stain, mouse brain sections at P16 were processed as described in ref. 27.

### *In vitro* electrophysiology

Brain coronal slices were prepared from *Efnb3*^+/+^ and *Efnb3*^*−*/*−*^ mice at P16. Brains were dissected quickly and chilled in ice-cold artificial cerebrospinal fluid containing (in mM): 119 NaCl, 2.5 KCl, 2.5 CaCl_2_, 1.3 MgCl_2_, 26 NaHCO_3_, 1 NaH_2_PO_4_ with 95% O_2_/5% CO_2_. Coronal brain slices from the region between the hippocampus and amygdala (400-mm thick) were prepared with a vibratome. After recovery, slices were placed in the recording chamber and continuously perfused with the artificial cerebrospinal fluid. Recording pipettes were pulled on a Flaming Brown P-80 PC Micropipette puller (Sutter Instrument, Novato, CA) filled with 3.5 M NaCl for extracellular recordings to achieve a resistance of 2–4 MΩ. Data were collected with an AxoClamp 2B recording amplifier (Molecular Devices, Union City, CA), FLA-01 Bessel filter unit (Cygnus Technologies, Delaware Water Gap, PA) and pCLAMP 9.0 software (Molecular Devices). Slices were stimulated using a bipolar concentric electrode (FHC, Bowdoin, ME) that was placed in ventral hippocampal CA1 pyramidal cells layer and connected with a stimulator (AMPI, Jerusalem, Israel). After a 15-min equilibration period, field potentials were recorded in the amygdala region. Each slice culture was recorded at 2-min intervals (2 min on, 2 min off) over a 20-min time period, for a total of 10 min of recorded activity.

### Statistical analysis

The results are presented as mean±s.e.m. Statistical differences were determined by Student's *t*-test for two-group comparisons or ANOVA followed by Dunnett's test for multiple comparisons among more than two groups.

## Additional information

**How to cite this article:** Zhu, X.-N. *et al*. Ephrin-B3 coordinates timed axon targeting and amygdala spinogenesis for innate fear behaviour. *Nat. Commun.* 7:11096 doi: 10.1038/ncomms11096 (2016).

## Supplementary Material

Supplementary Information Supplementary Figures 1-12

Supplementary Movie 1 Representative Efnb3+/+ mouse behavior in EPM trial at P16. The mouse spent almost all the testing time in close-arm of the elevated plus maze (EPM).

Supplementary Movie 2 Representative Efnb3-/- mouse behavior in EPM trial at P16. The mouse showed a longer time and a higher probability of open-arm entry in the EPM compared to the wild type Mice.

Supplementary Movie 3 Representative Efnb3+/+ mouse behavior in TMT trial at P16. The mouse presented many avoidance responses to TMT odor within one min.

Supplementary Movie 4 Representative Efnb3-/- mouse behavior in TMT trial at P16. The mouse presented less number of avoidance responses to TMT odor within one min compared to the wild type mice.
