# Supplementary material for: Ephrin-B3 coordinates timed axon targeting and amygdala spinogenesis for innate fear behaviour
Source: Nat Commun. 2016 Mar 24;7:11096. doi: 10.1038/ncomms11096 (PMC4820848; doi:10.1038/ncomms11096)
Supplement: Supplementary Information — Supplementary Figures 1-12 [file ncomms11096-s1.pdf]

Supplementary Figures

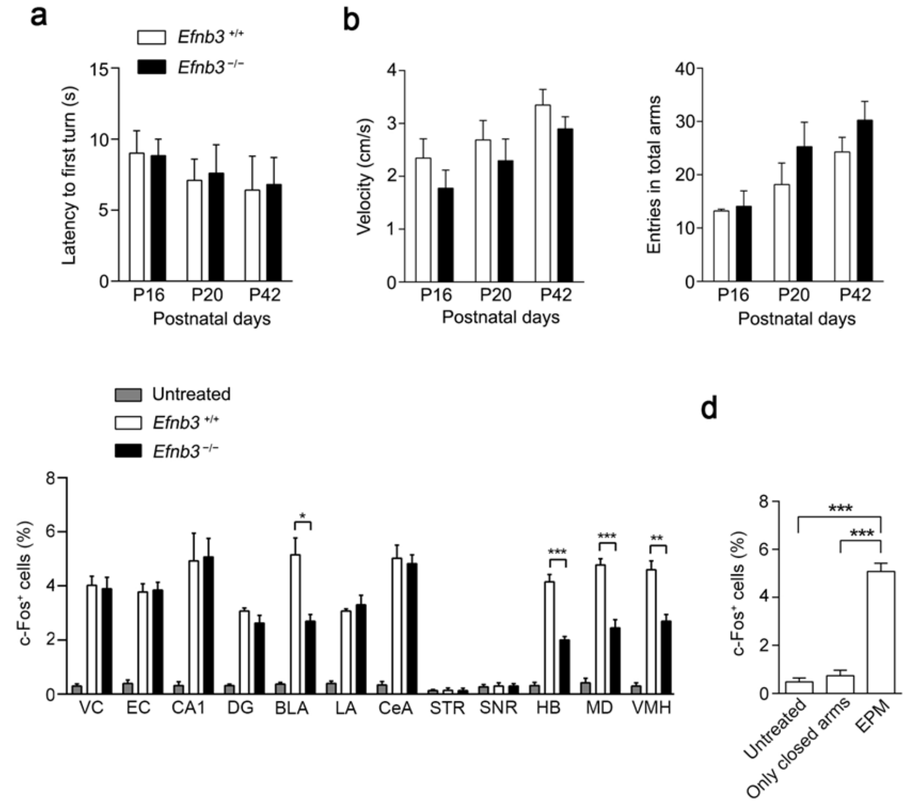

Supplementary Figure 1: Visual behavior and avoidance behavioral response in EPM trials. (**a**) Measures of visual behavior that performed the light avoidance behavior test. Time to first body turn away from the light for *Efnb3*$^{+/+}$ and *Efnb3*$^{-/-}$ mice at P16, P20 and P42. *n*=22 mice for *Efnb3*$^{+/+}$; *n*=24 mice for *Efnb3*$^{-/-}$; *P*>0.05, Student's t test. (**b**) Locomotor velocity (cm/s) and entries in total arms during the EPM test for *Efnb3*$^{+/+}$ and *Efnb3*$^{-/-}$ mice at P16, P20 and P42. *n*=22 mice for *Efnb3*$^{+/+}$; *n*=24 mice for *Efnb3*$^{-/-}$. *P*>0.05, Student's t test). (**c**) Quantification of activated c-Fos positive cells (normalized to total cell number) in visual cortex (VC), entorhinal cortex (EC), ventral hippocampal CA1 (CA1), dentate gyrus (DG), basolateral amygdala (BLA), lateral amygdala (LA), central amygdala (CeA), striatum (STR), substantia nigra (SNR), habenula nucleus (HB), mediodorsal thalamic nucleus (MD), ventromedial hypothalamus (VMH) in *Efnb3*$^{+/+}$ and *Efnb3*$^{-/-}$ mice following EPM or untreated trial at P16. *n*=4 mice per group. * *P*<0.05; ** *P*<0.01; *** *P*<0.001, one-way ANOVA. (**d**) Quantification of activated c-Fos positive cells in BLA in *Efnb3*$^{+/+}$ mice with untreated or only in closed arms or EPM trials at P16. *n*=4 mice per group. *** *P*<0.001, one-way ANOVA.

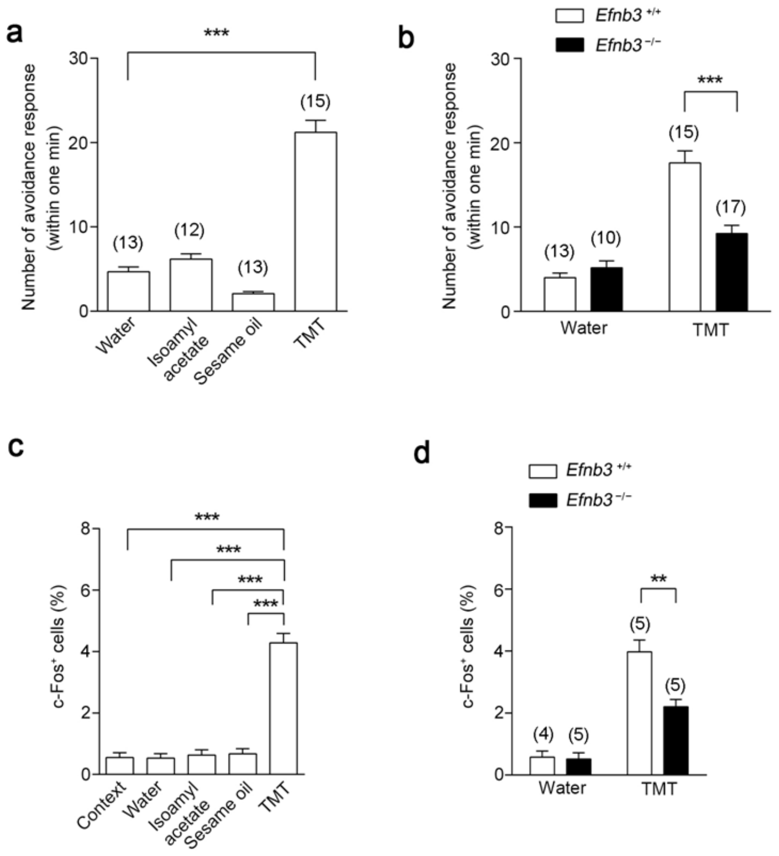

Supplementary Figure 2: Behavioral assay for innate responses to odors.

(**a**) Comparison of number of avoidance responses to non aversive and aversive odors (water, isoamyl acetate, sesame oil, TMT) within one min in wild-type mice at P16. (**b**) Avoidance responses to water and TMT within one min was quantified for *Efnb3*$^{+/+}$ and *Efnb3*$^{-/-}$ mice at P16. *n* values of mice used for (**a**) and (**b**) are indicated above each group bar.*** P<0.001, Student's *t* test. (**c**) Quantification of activated c-Fos positive cells in BLA of *Efnb3*$^{+/+}$ mice following different treatments. *n*=6 mice for each group. *** P<0.001, one-way ANOVA. (**d**) Quantification of activated c-Fos positive cells in BLA of *Efnb3*$^{+/+}$ and *Efnb3*$^{-/-}$ mice at P16 following water and TMT trials. *n* values of mice are indicated above each group bar. ** P<0.01, Student's *t* test.

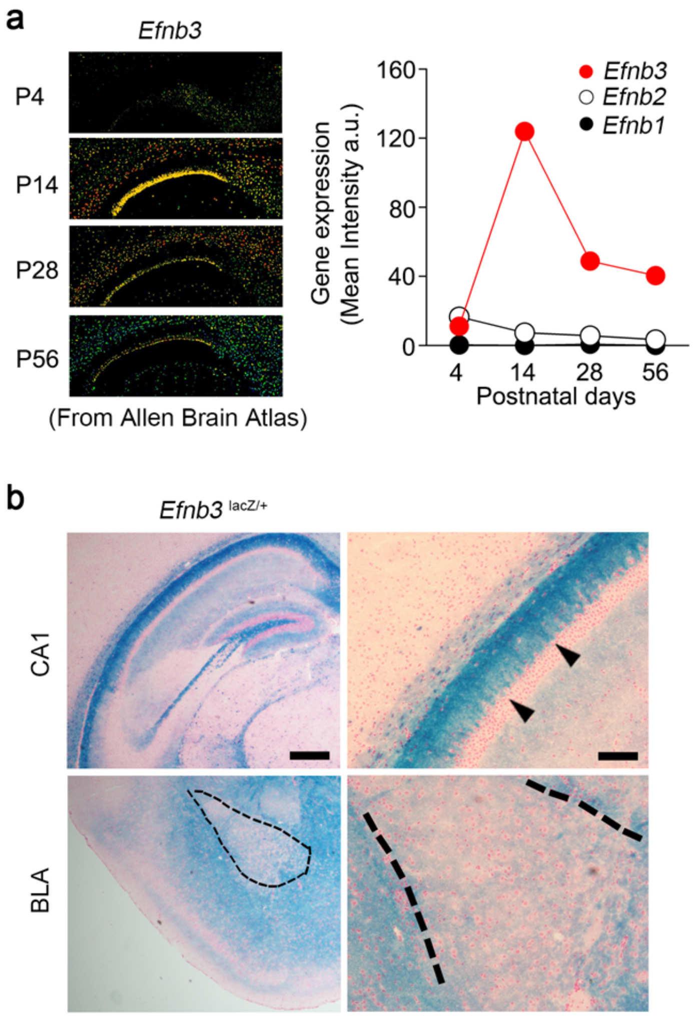

Supplementary Figure 3: Expression of eB3 in hippocampal CA1 area but not in amygdala. (**a**) Shown are data from the Allen Developing Mouse Brain Atlas mRNA in situ hybridization expression database. Typical images show *Efnb3* gene is expressed in CA1 pyramidal neurons at P4, P14, P28 and P56 in the left panel. Quantification of *Efnb1*, *Efnb2*, and *Efnb3* gene expression was performed in the right panel. The expression of Efnb3 in CA1 pyramidal neurons is specific and reaches a peak level at P14 during the developmental period, whereas *Efnb2* is expressed at low levels in CA1 neurons and *Efnb1* transcripts are undetectable in CA1 neurons at whole growth period. (**b**) Expression of eB3 visualized with X-gal staining of coronal sections for the eB3-β-gal fusion protein (blue) in *Efnb3*lacZ mice at P16. Structure of the hippocampus and amygdala was visualized with eosin counterstaining (red). Highly expression of eB3 was observed in dendrites field of CA1 region but not in basolateral amygdala. Scale bars: 500 μm in the left panel and 100 μm in the right panel.

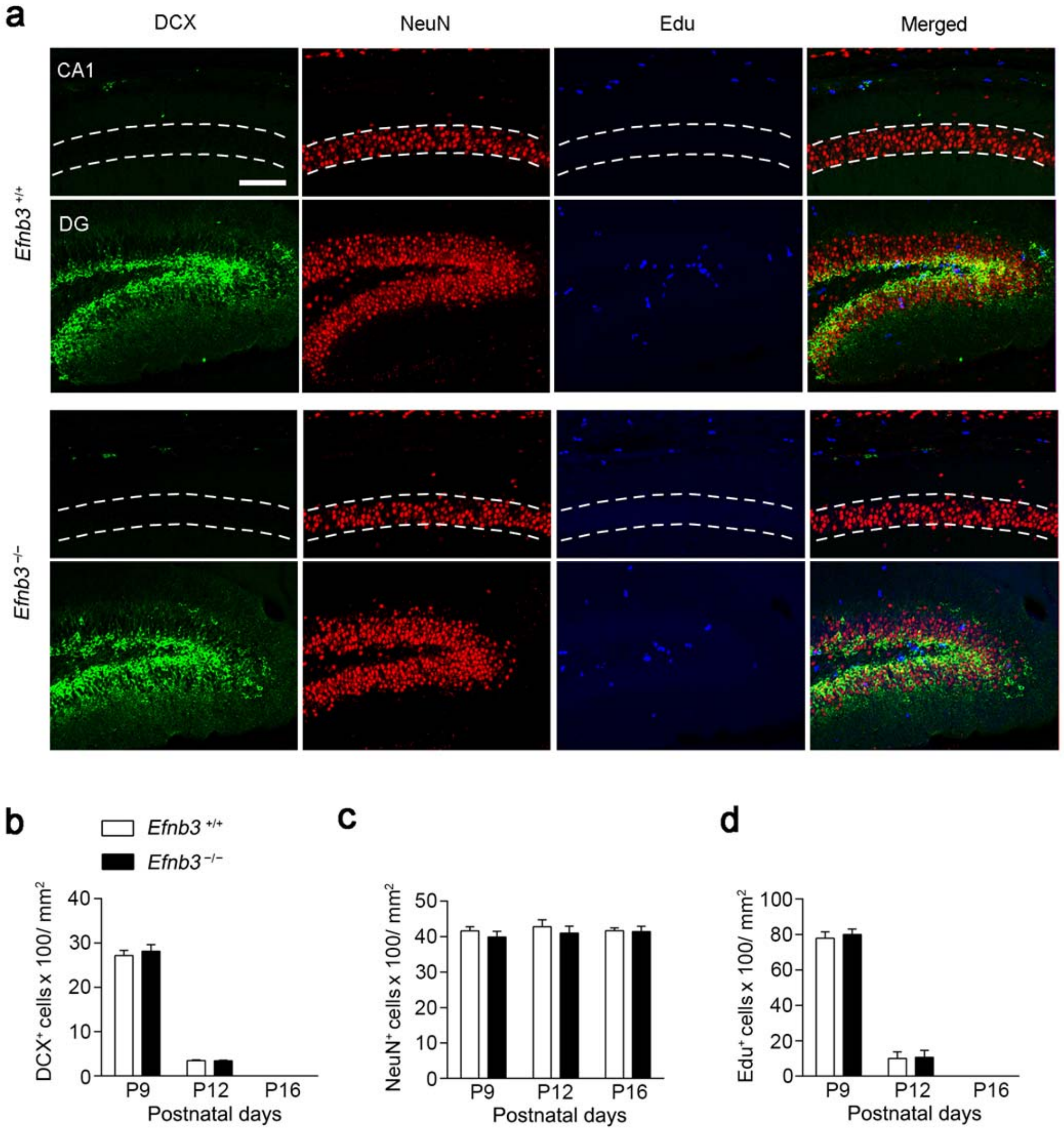

Supplementary Figure 4: Hippocampal neurogenesis and proliferation during postnatal days of *Efnb3*$^{+/+}$ and *Efnb3*$^{-/-}$ mice.

(**a**) Representative confocal images of CA1 and DG stained for doublecortin (DCX), NeuN, EdU (blue) in *Efnb3*$^{+/+}$ and *Efnb3*$^{-/-}$ mice at P16 following EdU injection 24 h ago. Scale bars: 100 μm. (**b**)-(**d**) Quantification of number of DCX positive cells (**b**) NeuN positive cells (**c**) and EdU positive cells (**d**) each mm^2 of CA1 region in *Efnb3*$^{+/+}$ and *Efnb3*$^{-/-}$ at P9, P12 and P16. *n*=3 mice per group, *P*>0.05 for all comparison between *Efnb3*$^{+/+}$ and *Efnb3*$^{-/-}$ mice, Student's *t* test.

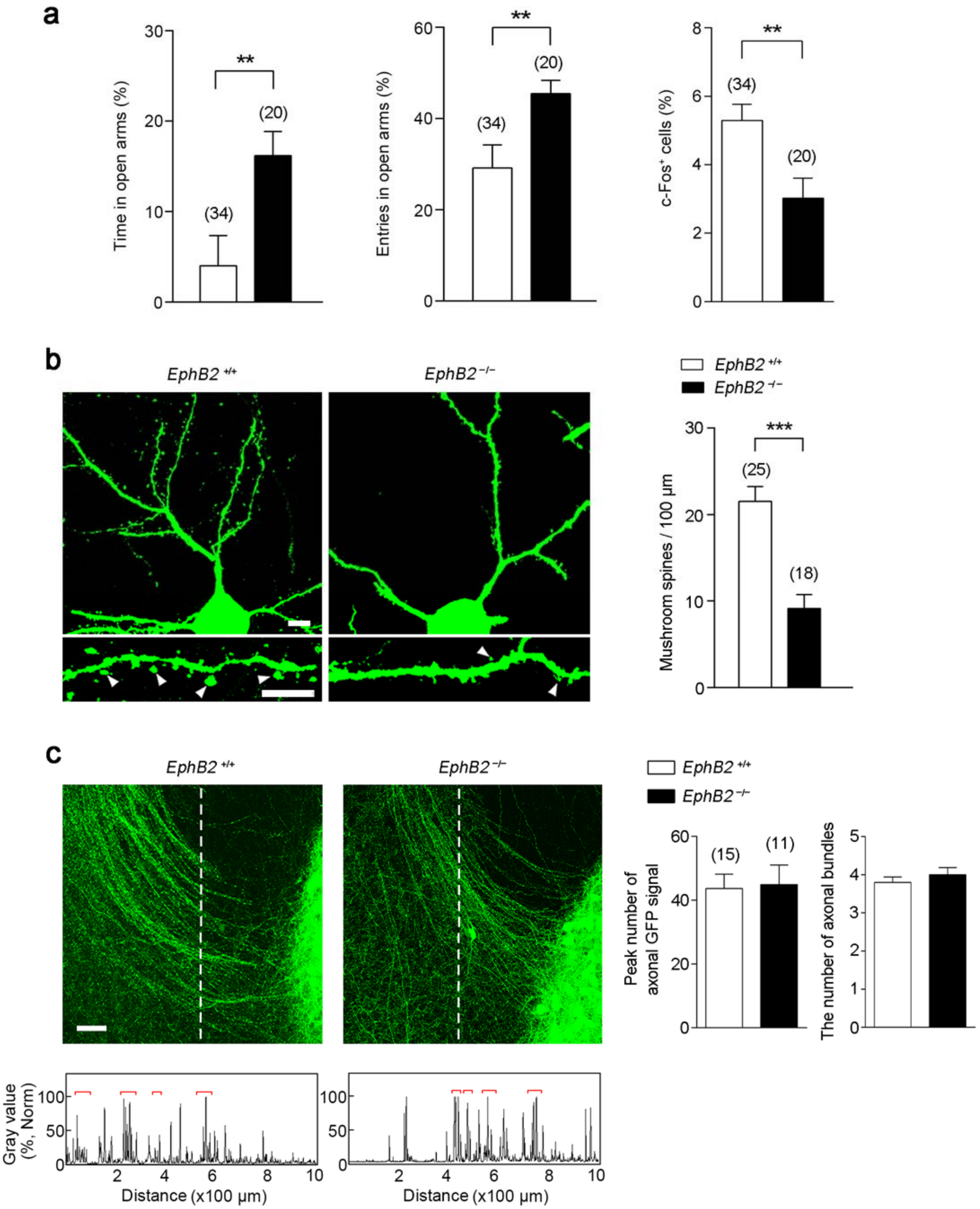

Supplementary Figure 5: EphB2 is required for defensive behavioral responses and spinogenesis in amygdala neurons but not for axon targeting toward BLA.

(**a**) *EphB2*$^{-/-}$ mice spent much more time and higher entry probability in the open arms than WT. The percentage of c-Fos positive neurons in BLA was decreased in *EphB2*$^{-/-}$ mutant at P16

following EPM behavior trial. *n* values of mice are indicated above each group bar. ** *P*<0.01, Student's *t* test. (**b**) EphB2 mutant caused low mushroom spine density in BLA neurons at P16. Scale bars, 10 µm. *n* values of neurons from 4 mice respectively are indicated above group bars. *** *P*<0.001, Student's *t* test. (**c**) The axonal bundles projected into amygdala in *EphB2*$^{-/-}$ mice at P16 were normal compared to *EphB2*$^{+/+}$ mice. Scale bar: 100 µm. The peak number of axonal GFP signal crossing the dotted line and fasciculated axonal bundles indicated with red square brackets in *EphB2*$^{+/+}$ and *EphB2*$^{-/-}$ mice at P16 were quantified. *n* values of brain slice from 5 mice per group are indicated above group bars. *P*>0.05 for the comparison between *EphB2*$^{+/+}$ and *EphB2*$^{-/-}$ mice, Student's *t* test.

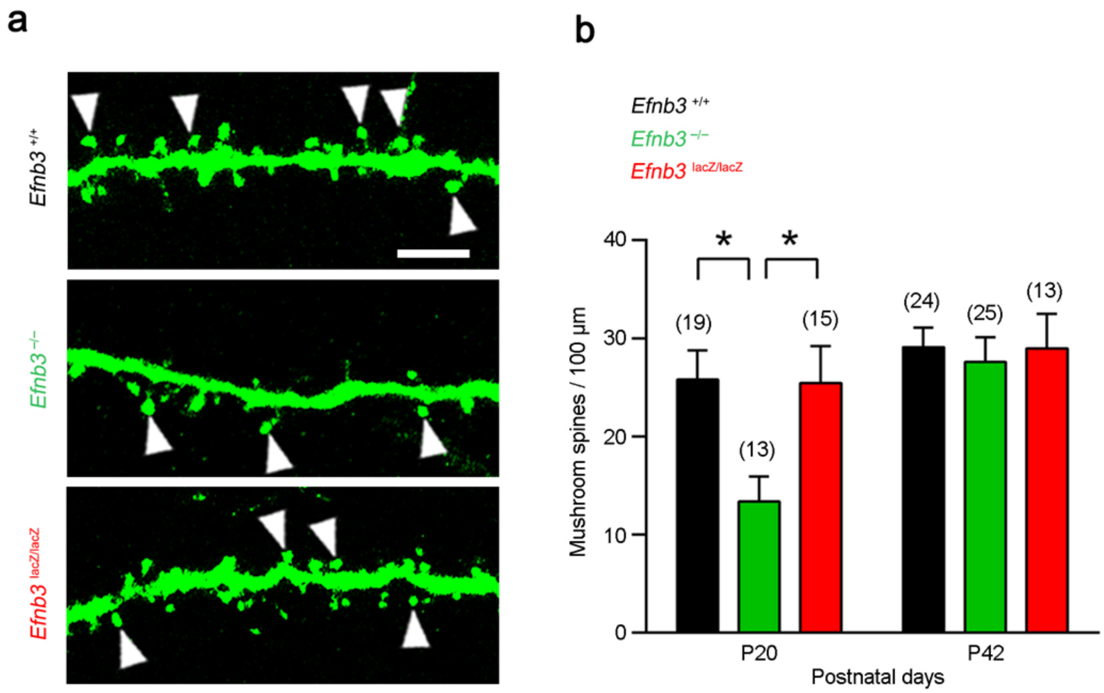

Supplementary Figure 6: Spine maturation of BLA neurons in *Efnb3*$^{+/+}$, *Efnb3*$^{-/-}$ and *Efnb3*$^{lacZ/lacZ}$ mice at P20 and P42.

(**a**) *Efnb3*$^{-/-}$ but not *Efnb3*$^{lacZ/lacZ}$ mutant mice showed fewer mushroom spines in BLA neurons at P20. Scale bar: 10 µm. (**b**) Quantification of mushroom spine density was performed in *Efnb3*$^{+/+}$, *Efnb3*$^{-/-}$ and *Efnb3*$^{lacZ/lacZ}$ mutant at P20 and P42. *n* values of neurons from 4-5 mice per group are indicated above group bars. * *P*<0.05, one-way ANOVA.

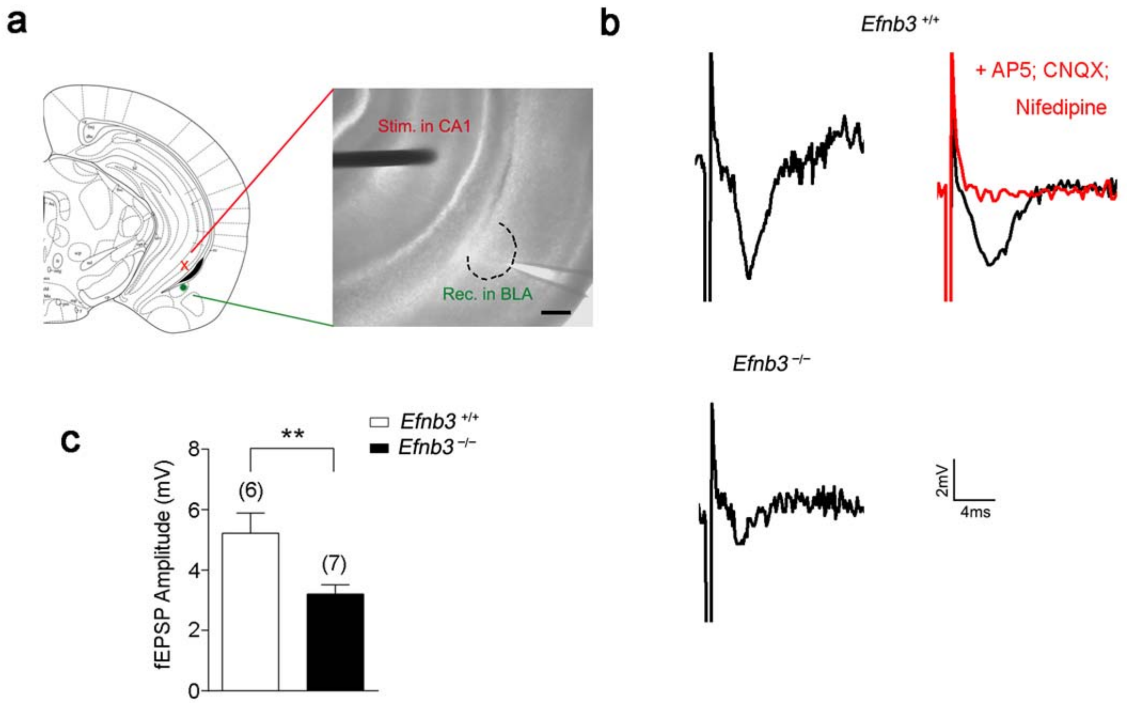

Supplementary Figure 7: Functional connection between ventral hippocampus and BLA in slice electrophysiology.

(**a**)A example for recording field excitatory postsynapic potential (fEPSP) in the ventral hippocampus-amygdala pathway. Stim. indicates stimulating electrode in ventral CA1, and Rec. indicates recording electrode in BLA. (**b**) fEPSP in amygdala region induced by stimulation of ventral CA1 pyramidal cells layer in *Efnb3*$^{+/+}$ and *Efnb3*$^{-/-}$ mice at P16. The fEPSP could be blocked by treatment of AP5 (50 µM), CNQX (20 µM) and Nifedipine (50 µM). (**c**) Quantification of amplitude of fEPSP in *Efnb3*$^{+/+}$ and *Efnb3*$^{-/-}$ mice at P16. *n* values of mice used are indicated above each group bar. ** *P*<0.01, Student's *t* test.

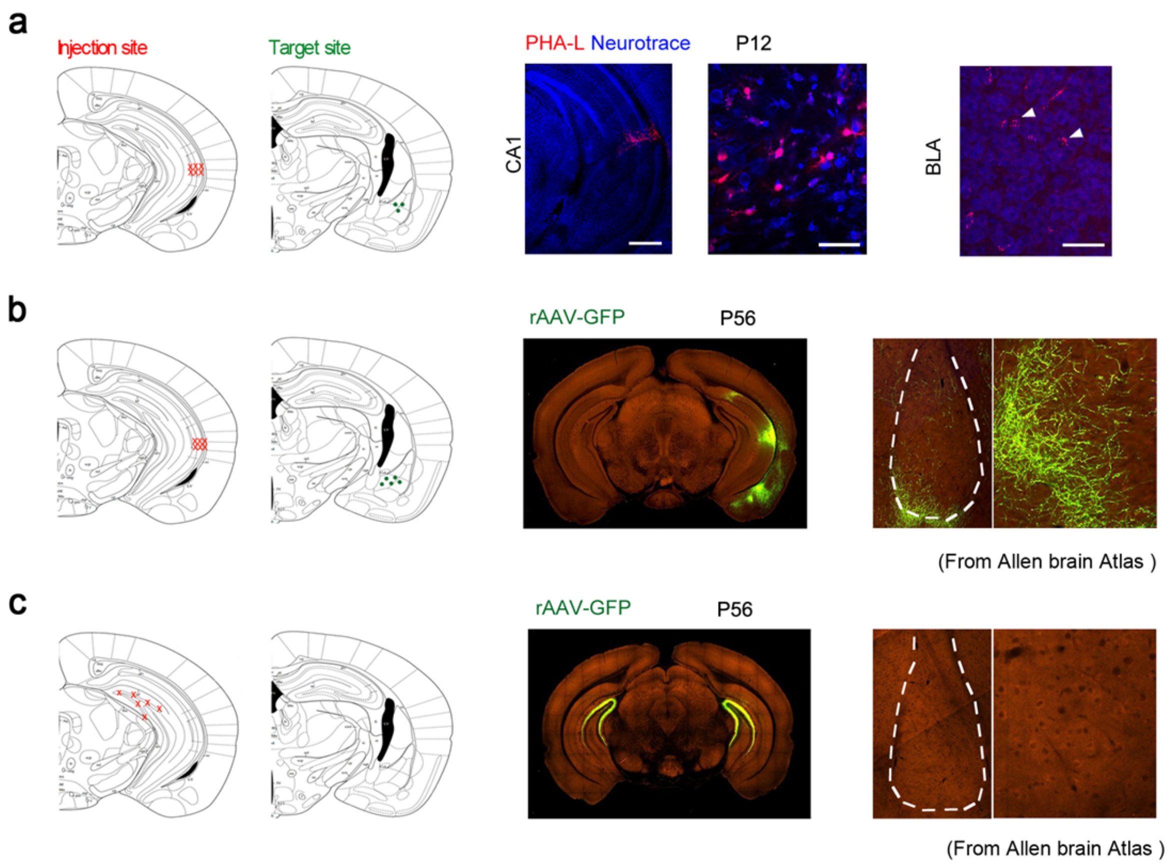

Supplementary Figure 8: Neural projection of hippocampus and amygdala neurons in juvenile and adult mice.

(**a**) Anterogradely labeled hippocampal-amygdala projecting neurons in hippocampal CA1 after an injection of tracing dye PHA-L into ventral CA1 at P12, PHA-L-labelled terminals (Red) from the ventral CA1 are visualized in BLA (arrowheads). Scale bar: 500 µm in the left panel and 100 µm in the right panel and 100 µm in the lower panel. (**b**) Anterogradely labeled hippocampal-amygdala projecting neurons in hippocampal CA1 after an injection of recombined-AAV virus (rAAV) into ventral CA1 at adult, rAAV-labelled terminals (green) from the ventral CA1 are visualized in BLA. Mode pattern of injection site in ventral CA1 (red markers) and its projecting areas in BLA (green circuit dots) at adult were shown in the left panels. (**c**) Anterogradely labeled hippocampal-amygdala projecting neurons in hippocampal DG after an injection of recombined-AAV virus (rAAV) into ventral DG at adult, there was not rAAV-labelled terminals from the DG in BLA. Mode pattern of injection site in ventral DG (red markers) and its possible projecting areas in BLA at adult were shown in the left panels. The immunofluorescence images of (**b**) and (**c**) are from Allen Brain Atlas.

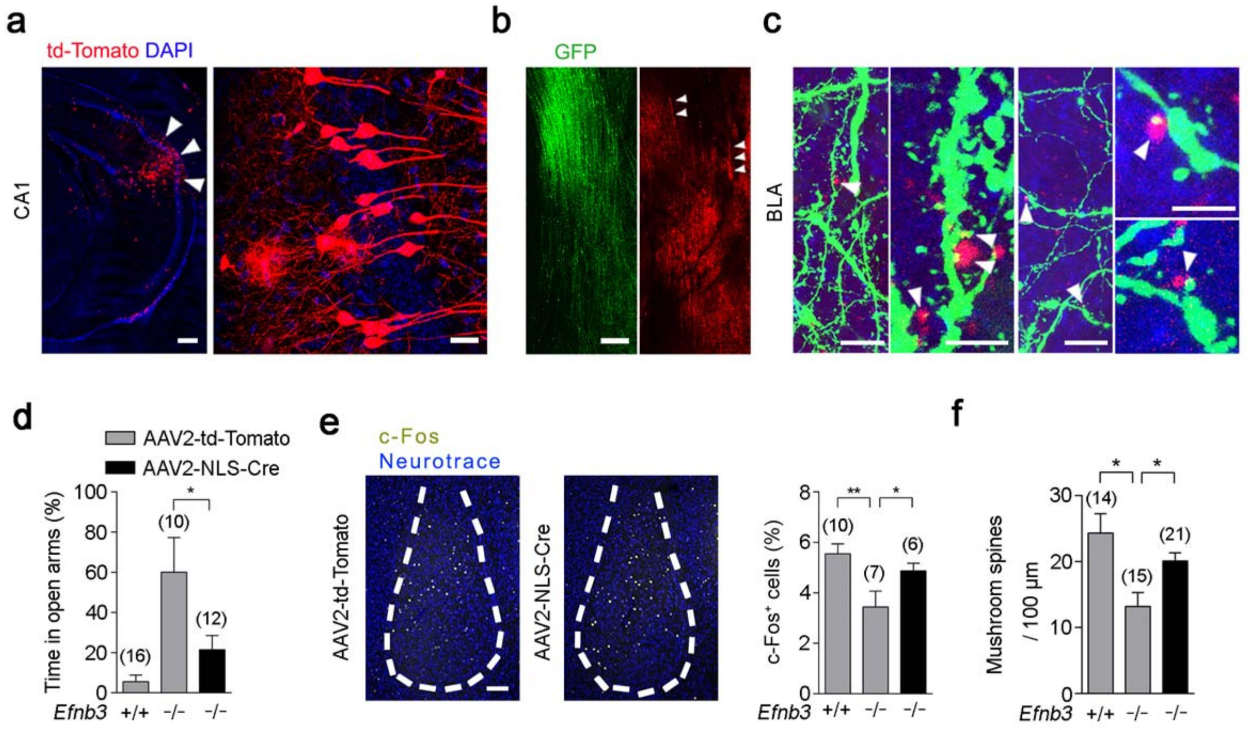

Supplementary Figure 9: Specific rescue of ephrin-B3 in CA1 neurons restores innate defensive reaction.

(**a**) Representative image of AAV2-NLS-Cre induced ephrin-B3 re-expression visualized with td-Tomato fluorescence in micro-injected hippocampal CA1 of *Efnb3*$^{-/-}$ mice. Scale bars: 200 µm in the left panel and 20 µm in the right panel. (**b**) The CA1 projected axons that labeled td-tomato were visualized in the position of axonal bundles labeled with Thy1-GFP. Scale bars: 20 µm. (**c**) The CA1 projected axon terminals formed synapses with dendritic spines of BLA neurons at P16. Scale bars: 10 µm in the left panel and 2.5 µm in the right panel. (**d**) AAV2-NLS-Cre injections that hit the CA1 area bilaterally significantly restored innate defensive behavior. The percentage of time in open arms was quantified. *n* values of mice are indicated above each group bar. *P<0.05, one-way ANOVA. (**e**) AAV2-NLS-Cre injections that hit the CA1 region bilaterally rescued expression of activated c-Fos in BLA at P16. Scale bar, 100 µm. The percentage of the cells with c-Fos expression in BLA was quantified in AAV2-td-Tomato or AAV2-NLS-Cre injected *Efnb3*$^{-/-}$ mice following behavioral trials. *n* values of mice used are indicated above each group bars. * P<0.05; ** P<0.01, one-way ANOVA. (**f**) AAV2-NLS-Cre injections that hit the CA1 region bilaterally rescued the defect mushroom spines in *Efnb3*$^{-/-}$ mice. *n* values of neurons from 4-6 mice per group are indicated above group bars. * P<0.05, one-way ANOVA.

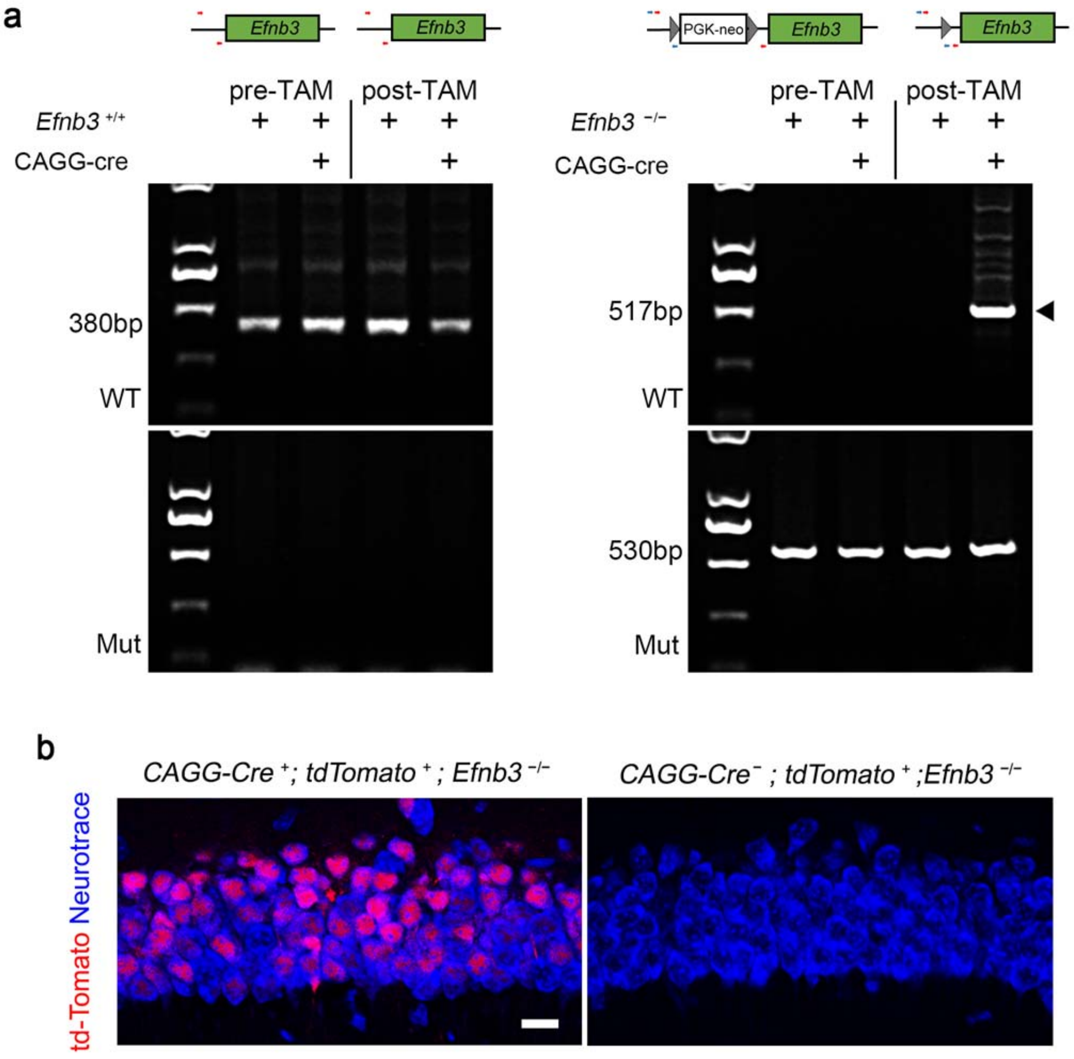

Supplementary Figure 10: Endogenous eB3 rescue by time-specific knock-in strategy in *Efnb3*$^{-/-}$ mutant.

(**a**) PCR genotyping shows that *Efnb3* is restored upon tamoxifen administration to induce Cre mediated excision of the *loxP*-flanked PGK neo cassette. Primers for *Efnb3*$^{+/+}$ (red arrows for WT) and *Efnb3*$^{-/-}$ mice (blue arrows for Mut) were used respectively to check the excision. *Efnb3* band (380bp) was not altered following tamoxifen administration in *CAGG-Cre*$^{+}$; *Efnb3*$^{+/+}$ mice (left panels). *Efnb3* could only be restored in *Cre*$^{+}$; *Efnb3*$^{-/-}$ mice after tamoxifen administration (517 bp band indicated with arrowhead, right panels). (**b**) Cre-mediated recombination in CA1 area was visualized in P16 hippocampal sections with td-Tomato fluorescence in *CAGG-Cre*$^{+}$; *td-Tomato*$^{+}$; *Efnb3*$^{-/-}$ mice but not in *CAGG-Cre*$^{-}$; *td-Tomato*$^{+}$; *Efnb3*$^{-/-}$ mice following tamoxifen administration at P12. Scale bar: 20 μm.

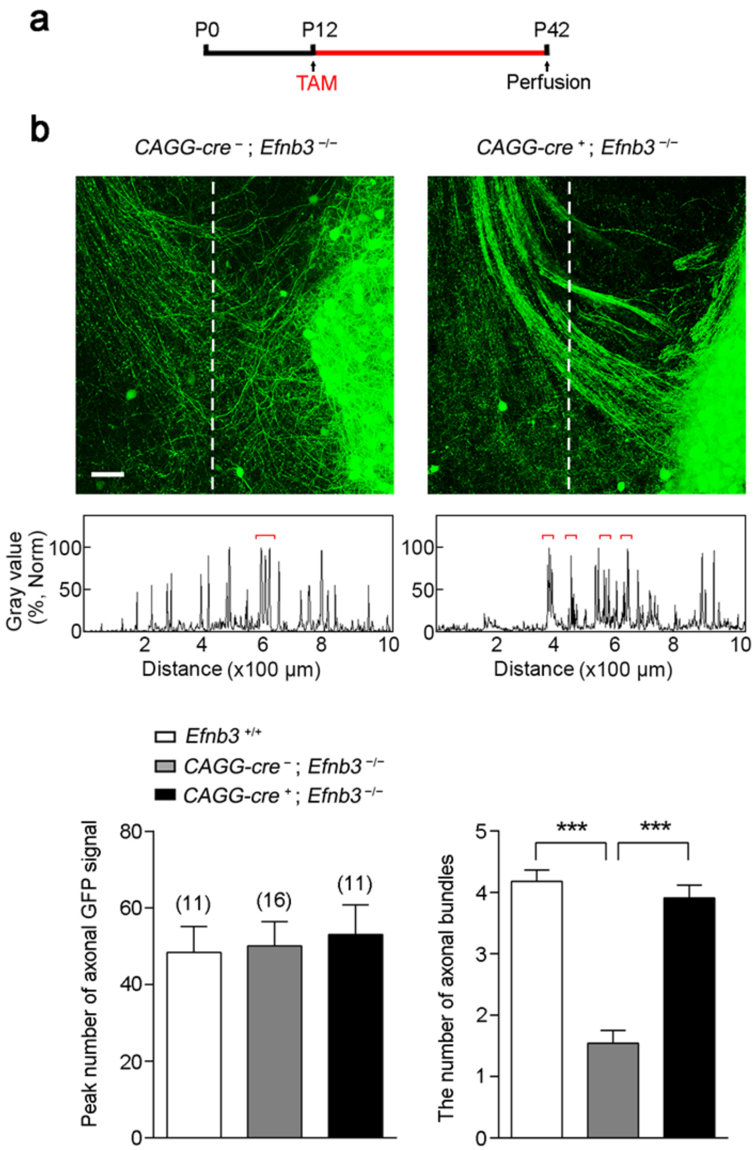

Supplementary Figure 11: Long-time eB3 rescue successfully restored the abnormal axonal pattern in *Efnb3*$^{-/-}$ mice.

(**a**) *CAGG-Cre*$^{+}$; *Efnb3*$^{-/-}$ mice were administrated with tamoxifen at P12 and perfused at P42. (**b**) Representative images showing axonal bundles projected into amygdala of *CAGG-cre*$^{-}$; *Efnb3*$^{-/-}$ and *CAGG-cre*$^{+}$; *Efnb3*$^{-/-}$ mice with Thy1-GFP background. Scale bars, 100 μm. Quantification of the peak number of axonal GFP signal crossing the dotted line and the fasciculated axonal bundles indicated with red square brackets in *CAGG-cre*$^{-}$; *Efnb3*$^{-/-}$ and *CAGG-cre*$^{+}$; *Efnb3*$^{-/-}$ mice at P42. *n* values of brain slices from 6-7 mice per group respectively are indicated above group bars. Mean ± s.e.m. *** *P*<0.001, one-way ANOVA.

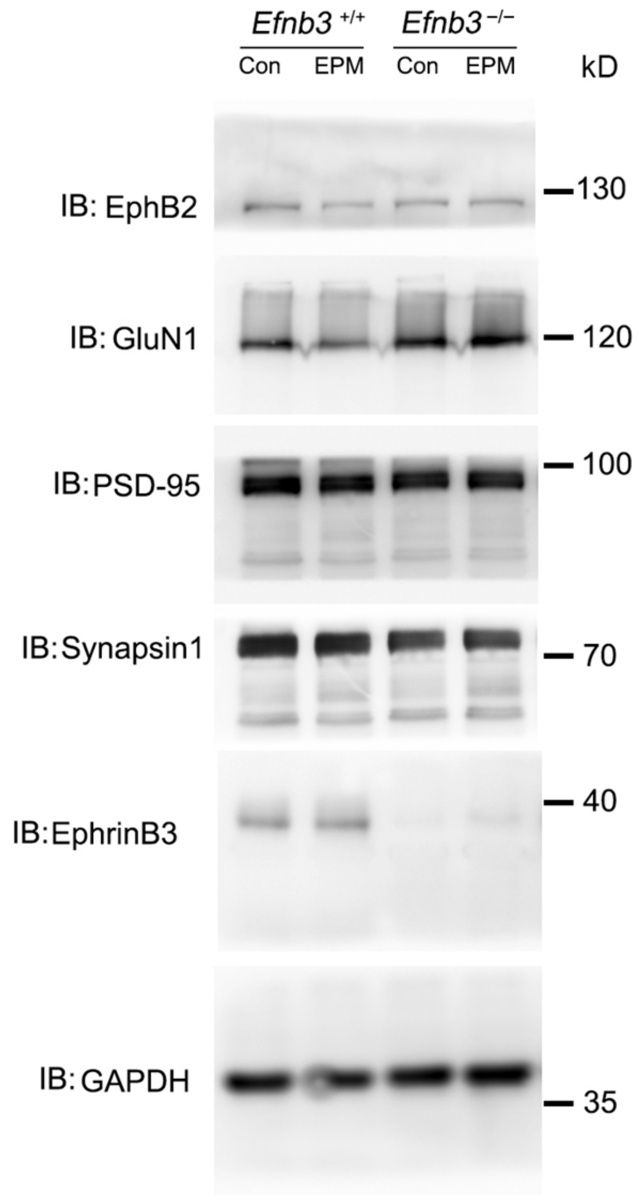

Supplementary Figure 12: Uncropped scans of western blots shown in Fig. 2c.
